# Sensory Input-Dependent Changes in Glutamatergic Neurotransmission- Related Genes and Proteins in the Adult Rat Trigeminal Ganglion

**DOI:** 10.3389/fnmol.2016.00132

**Published:** 2016-11-28

**Authors:** Julia Fernández-Montoya, Izaskun Buendia, Yasmina B. Martin, Javier Egea, Pilar Negredo, Carlos Avendaño

**Affiliations:** ^1^Departamento de Anatomía, Histología y Neurociencia, Universidad Autónoma de MadridMadrid, Spain; ^2^Instituto de Investigación Sanitaria, Hospital Universitario de La PrincesaMadrid, Spain; ^3^Departamento de Farmacología y Terapéutica, Instituto Teófilo Hernando, Universidad Autónoma de MadridMadrid, Spain; ^4^Departamento de Anatomía, Universidad Francisco de VitoriaMadrid, Spain

**Keywords:** primary afferents, input-dependent plasticity, enrichment, trimming, glutamate receptors

## Abstract

Experience-dependent plasticity induces lasting changes in the structure of synapses, dendrites, and axons at both molecular and anatomical levels. Whilst relatively well studied in the cortex, little is known about the molecular changes underlying experience-dependent plasticity at peripheral levels of the sensory pathways. Given the importance of glutamatergic neurotransmission in the somatosensory system and its involvement in plasticity, in the present study, we investigated gene and protein expression of glutamate receptor subunits and associated molecules in the trigeminal ganglion (TG) of young adult rats. Microarray analysis of naïve rat TG revealed significant differences in the expression of genes, coding for various glutamate receptor subunits and proteins involved in clustering and stabilization of AMPA receptors, between left and right ganglion. Long-term exposure to sensory-enriched environment increased this left–right asymmetry in gene expression. Conversely, unilateral whisker trimming on the right side almost eliminated the mentioned asymmetries. The above manipulations also induced side-specific changes in the protein levels of glutamate receptor subunits. Our results show that sustained changes in sensory input induce modifications in glutamatergic transmission-related gene expression in the TG, thus supporting a role for this early sensory-processing node in experience-dependent plasticity.

## Introduction

Glutamate is the most common excitatory neurotransmitter in both central and peripheral divisions of the nervous system. In sensory ganglia, including the trigeminal ganglion (TG), all types of neurons contain and release glutamate upon stimulation, not only from their central and peripheral axon terminals (McMahon et al., 1991; deGroot et al., 2000; Miller et al., 2011), but also from their cell bodies within the ganglia (Kung et al., 2013). Moreover, these neurons express molecules directly related to glutamate neurotransmission, such as ionotropic and metabotropic glutamate receptors (Sato et al., 1993; Ma and Hargreaves, 2000; Lu et al., 2002, 2003; Marvizon et al., 2002; Carlton and Hargett, 2007; Willcockson and Valtschanoff, 2008; Cahusac and Mavulati, 2009; Miller et al., 2011; Bouvier et al., 2015) and other molecules involved in the synthesis, transport and release of glutamate (Brumovsky, 2013; Kung et al., 2013).

Peripheral sensory nerve or tissue damage brings about rapid changes in glutamate receptors at various levels of the sensory pathways. Primary sensory neurons directly affected by axotomy or other nerve injuries display a multitude of cellular and molecular reactions that to a large extent reveal a self-protective and regenerative response (Fu and Gordon, 1997; Xiao et al., 2002; Lawson, 2005). Further upstream in the sensory pathway, however, neural changes reflect a combination of effects, those due to transsynaptic effects of deafferentation, and others resulting from the loss or distortion of the afferent input (Matthews, 1964; Panetsos et al., 2008; Herrera-Rincon et al., 2012). Both types of effects often, but not always, result in overlapping phenotypic changes in higher sensory centers. For example, whisker follicle removal in adult mice led to a long-lasting decrease in glutamic acid decarboxylase (GAD) immunoreactivity in the corresponding ‘barrels’ of the ‘barrel cortex’ (the division of the somatosensory cortex representing the mystacial vibrissae), which took months to revert (Welker et al., 1989). A similar, but shorter lasting effect was found after chronic whisker trimming (Akhtar and Land, 1991). The GLUR2 subunit of the alpha-amino-3-hydroxy-5-methyl-4-isoxazole propionic acid (AMPA) receptors was found to be differentially regulated in the visual cortex after monocular deprivation (Tropea et al., 2006). On the other hand, in the somatosensory adult cortex sustaining irreversible deafferentation by nerve transection, Mowery et al. (2013, 2015) found no differences with controls in the expression of GLUR2/3; however, this subunit was permanently elevated in the cuneate nucleus, the main target for large mechanosensitive fibers arising from the lost nerves. Furthermore, the activation of the *N*-methyl-D-aspartate (NMDA) receptor was required for plastic changes to occur in the visual cortex following monocular deprivation in adult mice (Sawtell et al., 2003).

The responses that sensory neurons display to changes in the signals incoming from the periphery may be distinguished from injury-associated reactions in these cells by manipulating input without damaging peripheral tissues, receptors and axon terminals. Sensory deprivation limited to active (haptic) touch in adult rodents is typically achieved by whisker trimming, and is known to result in a number of structural an functional changes in the cortex (Machin et al., 2006; Dolan and Cahusac, 2007). Besides, exposure of rats to an enriched environment has been often used as a contrasting paradigm to deprivation, to investigate sensory experience-dependent plasticity. This treatment induces genomic responses in the cortex that differ depending on the length of the exposure period. In an extensive microarray analysis, Rampon et al. (2000) found that 100 transcripts, many of which were related to synaptic transmission and neuronal structure, changed at least 1.5-fold in the whole neocortex of adult mice after 2 weeks exposure to enrichment. Similar changes in expression were found when the exposure to enrichment was limited to 3–6 h, but the genes involved were fewer, and most of them were important in coding proteins involved in nucleic acid and protein synthesis, regulation and processing. Also, after brief periods of exposure to enrichment, Brain-derived neurotrophic factor (Bdnf) and several related immediate-early response genes (Immediate Early Genes, IEG) are typically upregulated in the rat barrel cortex (Bisler et al., 2002; Staiger, 2006; Valles et al., 2011).

In contrast with the cortex, little is known about the long-term effects of sensory input modifications on the gene expression in lower centers of somatosensory processing. Nevertheless, experience-dependent changes at the earlier steps of sensory processing are relevant to understand the structural and functional plasticity found at higher levels of the pathways, in particular the cortex (Panetsos et al., 1995; Kaas et al., 1999; Negredo et al., 2009; Oberlaender et al., 2012; Martin et al., 2014). Given the importance of glutamatergic neurotransmission in the somatosensory system, we set out to investigate the mRNA and protein expression of glutamate receptor (AMPA, NMDA, and metabotropic) subunits and other molecules closely related to glutamate neurotransmission in the TG of young adult rats under three conditions: (1), naïve, (2), long-term exposure to sensory-enriched environment, and (3), repeated unilateral whisker trimming.

## Materials and Methods

### Experimental Animals

Young adult (10–12 weeks old) male Sprague-Dawley rats (*n* = 34) from our own colony, originating from Harlan (Harlan Iberica, Barcelona, Spain) were used. All animal procedures were approved in advance by the Ethical Committee of the Autonoma University of Madrid, in accordance with European Community’s Council Directive 2010/63/UE. Every effort was made to minimize the suffering of the animals, as well as the number of animals used.

The animals were divided into three groups: (1) Control Group (C, *n* = 12) included animals that were kept under standard housing conditions until the end of the experiment. (2) Trimming Group (T, *n* = 10), rats that were subjected to unilateral deprivation of active (haptic) touch by cutting all whiskers in the right side every 2–3 days for 7 weeks. Whiskers were trimmed to within 1 mm on hand-held awake animals, so that regrown vibrissae never exceeded 3 mm (Ebner, 2005). Extreme care was taken to avoid plucking the vibrissae or damaging the skin, so that deprivation was achieved without damaging trigeminal receptors and pathways. (3) Enriched Environment Group (E, *n* = 12), rats that were exposed to a sensory-enriched environment 4 h/day, 5 days/week for 7–8 weeks. In this “enriched” condition groups of eight animals were placed in a large cage (100 cm × 80 cm × 60 cm) with various beddings and a set of toys, ramps, tubes and other natural and artificial objects of different textures, which were changed every 5th day (four animals of the second group were used for a separate study). Not more than two littermates from the same dam were used in each experimental group. All rats were housed under standard colony conditions (Four rats per cage). Food and water were supplied *ad libitum*, and the animals were kept under a reversed 12:12 h dark/light cycle.

### Tissue Samples

The animals were anesthetized (Dolethal, 50 mg/kg i.p.), decapitated and the TG of both sides were rapidly excised by sectioning the trigeminal root and the three trigeminal branches. For the mRNA study four ganglia from each side were pooled, and each pool was separately placed in an ice-cold stabilization reagent (RNAlater^®^, Qiagen, CA, USA) and frozen at -80°C until mRNA extraction. For the protein analysis, the TG from each side was collected separately (*n* = 8 × 2 ganglia from Groups C and E, and *n* = 6 × 2 ganglia from Group T); samples were prepared by homogenizing in ice-cold lysis buffer [20 mM Tris, pH 7,5, 1% NP-40, 10% glycerol, 137 mM NaCl, 20 mM NaF, 1 mM NaPPi, 1 mM Na3VO4, 1 μg/ml leupeptin, 1 mM PMSF and protease inhibitors cocktail (COMPLETE Mini, Roche)]. The protein concentration in the samples was determined using the Pierce^TM^ BCA Protein Assay Kit (Thermo Fischer Scientific Inc., GA, New York, NY, USA) following manufacturer’s instructions.

### Microarray, Labeling, and Hybridization

Details on the development of the RT^2^ Profiler^TM^ PCR Arrays (Qiagen, Valencia, CA, USA) and the list of the genes included in the array are available at http://www.sabiosciences.com/rt_pcr_product/HTML/PARN-126Z.html. The array includes 84 genes involved in plasticity representing IEGs, late response genes, proteins involved in long term potentiation (LTP) and long term depression (LTD), cell adhesion molecules, extracellular matrix and proteolytic processing molecules, CREB cofactors, neuronal receptors, postsynaptic density proteins and others. According to the manufacturer, controls are also included on each array for genomic DNA contamination, RNA quality, and general PCR performance. The PCR Array System demonstrates high reproducibility with strong correlations across technical replicates, lots, and instruments with average correlation coefficients > 0.99 ensuring reliable detection of differences in expression between biological samples and high specificity with high quality input RNA. RNA extraction, labeling, and hybridization were performed according to the protocol provided by the manufacturer. In order to reduce biological variation (Kendziorski et al., 2005) pools of four whole TG from each experimental group and side were prepared to extract the mRNA.

Samples were disrupted using TissueLyser system and mRNA was extracted with RNeasy^®^ Mini Kit (Qiagen, Valencia, CA, USA) in a Qiacube robotic work station. The purity of the RNA was determined with de ratio *A*_260_/*A*_280_ which was greater than 1.8 in all cases, the absorbance ratio was calculated using a Nanodrop ND-1000 Spectrophotometer (Thermo Fischer Scientific Inc., GA, New York). The ribosomal band integrity was analyzed with an Agilent 2100 Bioanalyzer (Agilent Technologies, Santa Clara, CA, USA) and the RNA Integrity Number (RIN) was greater than 7 in all cases. Total RNA (50 ng) was retrotranscribed using RT^2^ First Strand kit (Qiagen, Valencia, CA, USA). The expression profile of the genes was determined using a 384-well format Synaptic Plasticity RT^2^ Profiler^TM^ PCR Array (SaBiosciences, USA) according to the manufacturer’s instructions. In addition to the 84 target genes mentioned above, the array included six housekeeping genes and three RNAs as internal controls. qPCRplates were run on an ABI 7900HT qPCR instrument (Thermo Fischer Scientific Inc., GA, New York) equipped with SDS 2.3 software, using RT^2^ SYBR Green/ROX qPCR master mix (Applied Biosystems, UK).

### Western Blot

Proteins (60 μg) from the lysates were separated by sodium dodecyl sulfate-polyacrylamide gel electrophoresis and transferred to Inmobilon-P membranes (Millipore Iberica SA, Madrid, Spain). Then, membranes were incubated with the following antibodies: rabbit polyclonal anti-mGLUR3 (1:500, Abcam, Cambridge, MA, USA), mouse monoclonal anti-GLUR2 (1:500, NeuroMab, Davis, CA, USA), mouse monoclonal anti-NMDAR2B (1:500, NeuroMab, Davis, CA, USA), mouse monoclonal anti-PICK1 (1 μg/ml, Abcam, Cambridge, MA, USA), rabbit polyclonal anti- PSD95 (1:1000, Cell Signaling Technologies, Beverly, MA, USA), and mouse monoclonal anti-β3-tubulin (1:10000, Calbiochem, La Jolla, CA, USA). Appropriate peroxidase-conjugated secondary antibodies (1:10000) were used and chemoluminiscence was measured by Advance Western-blotting Detection Kit (GE Healthcare, Barcelona, Spain). Finally, optical density was quantified using Scion Image^®^ Alpha 4.0.3.2 program.

### Data Analysis

Array data analysis was performed using the PCR Array Data Analysis Web Portal^1^). The quantification was done using the ΔΔ*C*_T_ method. Among the housekeeping genes present in the array we selected one (Hprt1), which was stably expressed in both sides of the three groups, to perform data normalization. For a gene to be considered differentially expressed, we arbitrarily selected a cutoff of twofold change up or down in the expression ratio. Additionally, we set the RT-PCR cycle threshold at 30 or less to ensure reliability, according to the manufacturer’s instructions. Following this conservative criterion, 18 genes out of the 84 genes present in the array were excluded from further analysis. Western blot data analysis was performed using GraphPad Prism 5.03 statistical software. Paired or unpaired *t*-tests (two-tailed) were applied for comparison between sides in the same group or between the control group and each of the input-altered groups, respectively. Comparison between sides in the same group was performed taking the right side as 100%, and using the relative values, as percentages, for the left side. Statistical significance was set at a *p*-value < 0.05.

## Results

### mRNA Expression in the TG

#### Controls (Group C)

**FIGURE 1:**
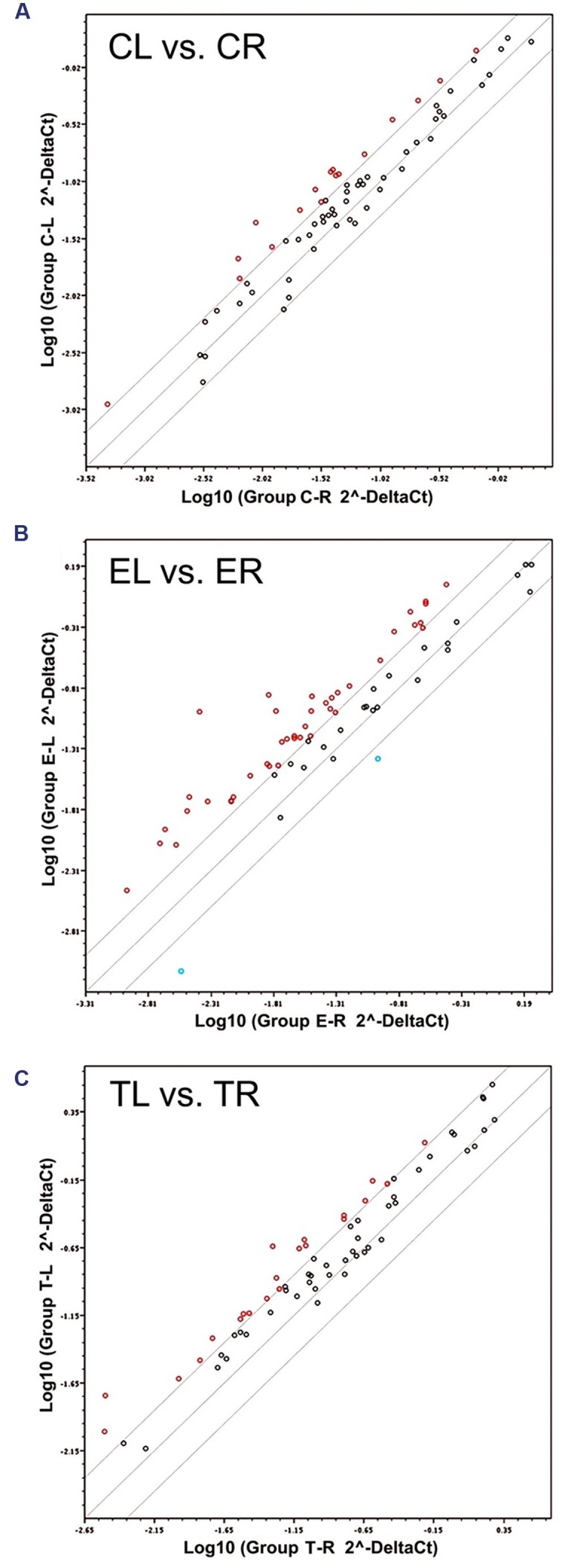
**Scatter plots of the 66 target mRNAs expression in trigeminal ganglia (TG).** The expression profile is plotted as log10 (2ˆ-Delta Ct) in the left and right TG for the Control **(A)**, Enriched **(B)**, and Trimmed **(C)** groups. Values on the diagonal midline represent equal expression in both sides. Lines above and below the midline indicate the twofold cut-off boundaries of expression; colored circles above (red) and below (blue) these lines identify over- and under-expressed genes, respectively.

**FIGURE 2:**
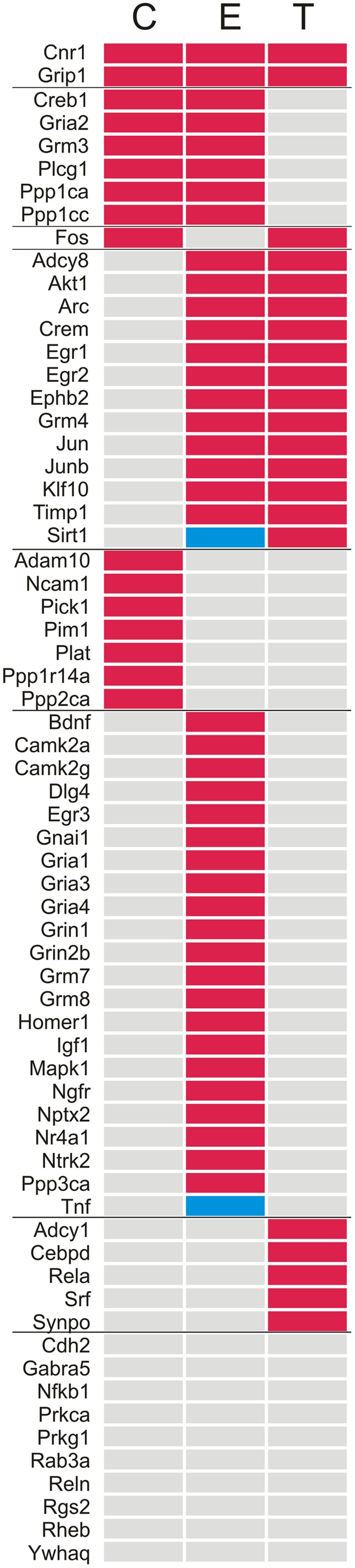
**List of the genes sorted according to the presence or absence of ≥2-fold up (red) or down (blue) differences in expression in the left vs. the right TG.** C, Control; E, Enriched; T, Trimming.

Of all 66 target genes, 16 showed at least a twofold difference in expression between the right and the left TG in control animals. The differentially expressed genes included immediate early response genes, genes involved in LTP or LTD, and genes coding for cell adhesion molecules and neuronal receptors. In all cases of differential expression, the levels were higher in the left TG (**Table 1**; **Figures 1A** and **2**).

**Table 1:** List of genes studied in the array.

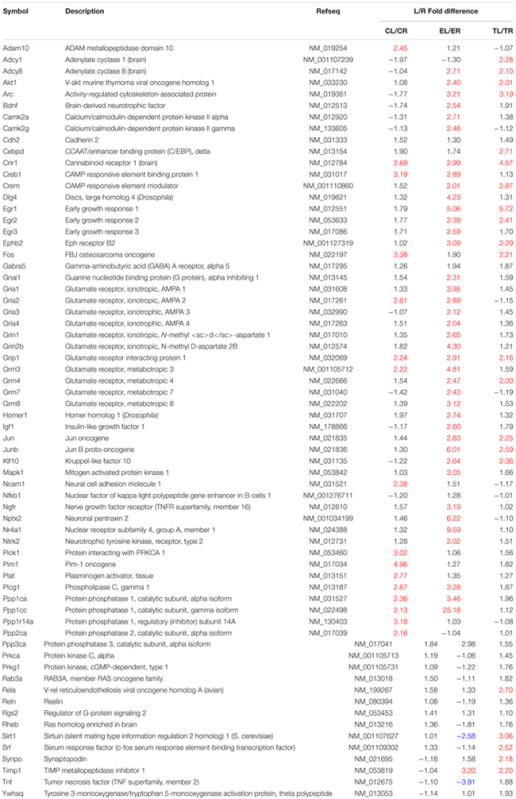

*The three columns on the right show the fold differences between sides in the TG of control, enriched and trimming groups. Values in red and blue emphasize ≥2-fold up or down changes in the expression ratio, respectively. CL, control left; CR, control right; EL, enriched left; ER, enriched right; TL, trimming left; TR, trimming right.*

#### Environmental Enrichment (Group E)

In animals chronically exposed to an enriched environment, the number of genes showing differential expression between sides (43/66) nearly tripled compared to controls. As in controls, a large majority (41/43) displayed higher expression in the left side (**Table 1**; **Figures 1B** and **2**), and these comprised, among others, a wide range of immediate early response genes, and LTP- and LTD-related genes (including several genes related to glutamatergic neurotransmission).

#### Trimming Group (Group T)

Compared to the left–right asymmetries found in controls, sustained whisker trimming on the right side led to an increase in a number of genes that were overexpressed in the left TG compared to the right (21/66). Fourteen of these genes overlapped with those overexpressed on the left in Group E, while just three genes that were overexpressed on the left in Group T (Cnr1, Fos, and Grip1) overlapped with those upregulated on the left in Group C (**Table 1**; **Figures 1C** and **2**).

### Glutamate Transmission-Related Genes: mRNA Expression

Out of the 66 genes analyzed, 15 coded for proteins directly involved in glutamatergic neurotransmission, which was the main focus of this study. Four corresponded to subunits 1–4 of the AMPA receptor (Gria1-4); another two referred to subunits 1 and 2b (Grin1, Grin2b) of the NMDA receptor; and four coded for four of the eight major types of metabotropic receptors, mGluR3,4,7,8 (Grm3, Grm4, Grm7, and Grm8). Furthermore, five other genes were selected as associated to glutamate neurotransmission (Dlg4, Grip1, Homer1, Nptx2, and Pick1), because of the involvement of the proteins they code in the clustering, anchoring, removal, and/or recycling of the glutamate receptor subunits on the cellular membrane. All of these genes showed at least a twofold difference in expression between sides in one or more groups of rats.

#### Asymmetries in Controls

Two genes, each one coding for one subunit of AMPA (Gria2) and metabotropic (Grm3) receptors showed higher expression in the left TG, as did Grip1 and Pick1 (**Table 1**).

##### Changes associated to exposure to an enriched environment

Except for Pick1, the same genes that showed higher expression in the left TG in controls appeared up-regulated in Group E. In addition, an increased expression in the left side of nine more genes (Dlg4, Gria1, Gria3, Gria4, Grin2b, Grm4, Grm7, Grm8, and Nptx2) coding for several glutamate receptor subunits, PSD95, and a neuronal pentraxin was noticed after enrichment (**Table 1**).

Side-by-side comparisons between Groups E and C revealed a general trend toward down-regulation in the right TG and little change in the left, suggesting that larger asymmetries after enrichment were mostly due to a decreased expression in the right, rather than to an up-regulation in the left TG (**Table 2**). Exceptions to this pattern were Pick1, which was up-regulated in the right ganglion, and Dlg4 and Gria1, which were up-regulated in the left TG.

**Table 2:** List of glutamate transmission-related genes studied in the array.

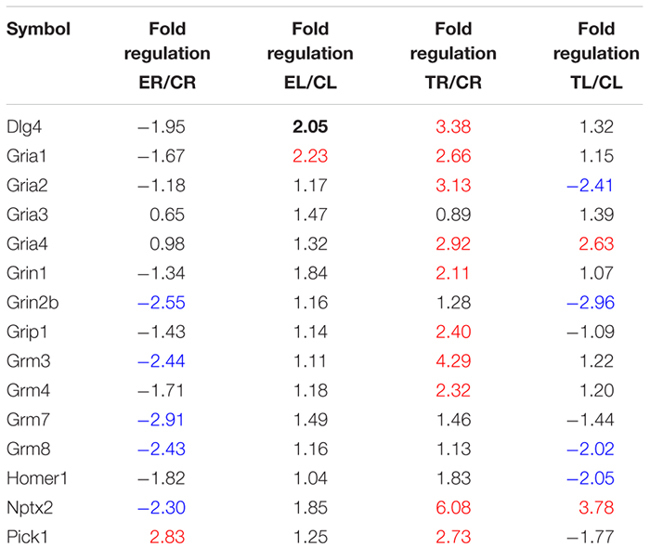

*The four columns show fold differences in expression for each side between groups, using the control group as the reference. Values in red and blue highlight ≥2-fold up or down changes in the expression ratio, respectively.*

##### Changes resulting from repeated whisker trimming in the right side

On the whole, the lateral asymmetries in gene expression found in controls disappeared after repeated trimming of the whiskers in the right side, with the only exception of Grip1. In addition, one gene that showed no differences between sides in controls (Grm4) was up-regulated in the left TG after trimming (**Table 1**).

Most of these changes with respect to controls resulted from an extensive up-regulation of gene expression in the right TG, that is, the side where chronic sensory deprivation took place, combined with a less marked down-regulation in the left ganglion (**Table 2**). The up-regulation on the right involved genes coding for various AMPA, NMDA, and metabotropic receptor subunits, as well as Dlg4, Grip1, Nptx2, and Pick1. On the left, three genes, each one coding for one subunit of AMPA (Gria2), NMDA (Grin2b), and metabotropic (Grm8) receptors, as well as Homer1, were down-regulated, and just two (Gria4 and Nptx2) were up-regulated.

### Glutamate Transmission-Related Protein Levels in the TG

**FIGURE 3:**
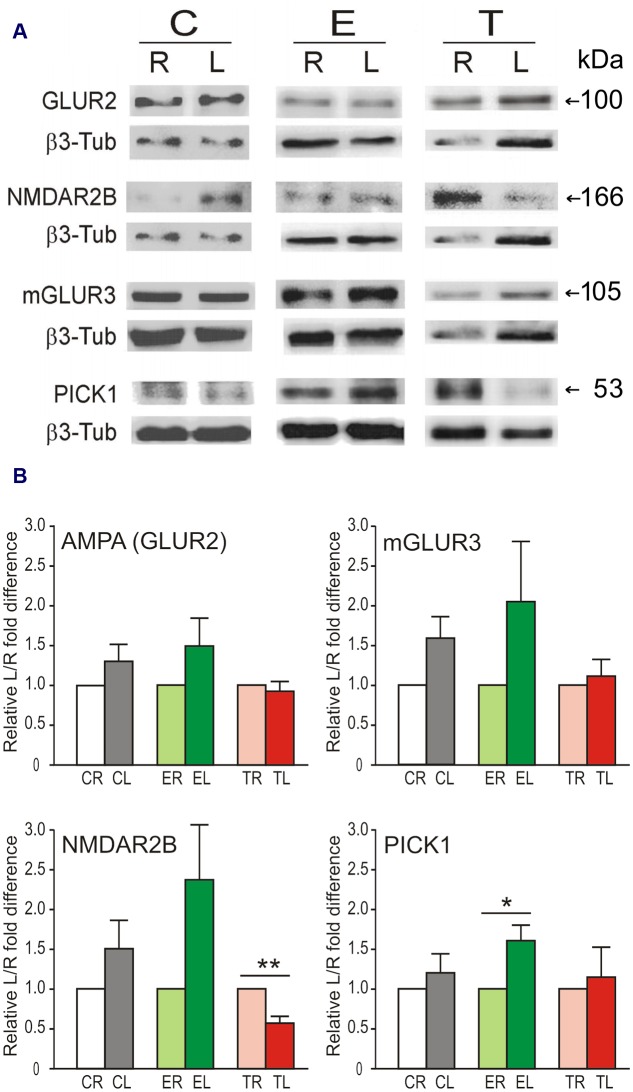
**Lateral differences in protein levels in the TG.** **(A)** Representative immunoblots showing protein levels of GLUR2, NMDAR2B, mGLUR3, and PICK1 in left (L) and right (R) sides of the TG in the same animal from Control, Enriched, and Trimmed groups. β3-tubulin was used as loading control. **(B)** Variation in protein expression level is given taking the right side as 100%. Left over right side differences in protein levels of GLUR2, NMDAR2B, mGLUR3, and PICK1 in the TG. Error bars indicate SEM. ^∗^*p* < 0.05, ^∗∗^*p* < 0.01 compared with relative control groups; student’s paired two-tailed *t*-test.

To assess the possible correlation of changes in mRNA levels with protein levels, four proteins coded by genes of various types that displayed relevant lateral differences in expression and changes after manipulation of sensory input were selected for analysis. In general, the levels of these proteins (subunit GLUR2 of the AMPA receptor, NMDAR2B, mGLUR3, and PICK1) appeared higher in the left TG than in the right TG in controls and enriched animals (**Figure 3**). Statistically, however, only in a few cases did these differences reach statistical significance or tendency (paired two-tailed *t*-tests; Group C: mGLUR3, *p* = 0.072; Group E: NMDAR2B, *p* = 0.087, PICK1, *p* = 0.022). In contrast, Group T showed significantly lower levels of NMDAR2B (*p* = 0.003) in the left TG. PSD95 protein was not detected in TG.

**FIGURE 4:**
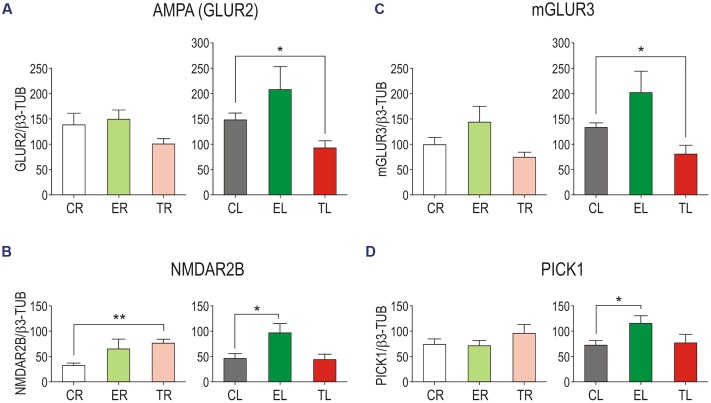
**Side-by-side comparison between groups of GLUR2 (A)**, NMDAR2B **(B)**, mGLUR3 **(C)**, and PICK1 **(D)** protein levels in the TG. Error bars indicate SEM. ^∗^*p* < 0.05, ^∗∗^*p* < 0.01; student’s unpaired two-tailed *t*-test.

Side-by-side comparisons between groups (**Figure 4**) showed a trend of increased levels of all four proteins in the left TG in Group E compared with Group C. This difference reached statistical significance for NMDAR2B (unpaired two-tailed *t*-tests for same side comparisons; *p* = 0.034), and PICK1 (*p* = 0.030). After sustained whisker trimming, in contrast, GLUR2 and mGLUR3 markedly diminished in the left TG compared to controls (*p* = 0.026 and *p* = 0.016, respectively). Group T also exhibited changes in protein levels in the deprived side, more notably a significant increase in the NMDAR2B subunit (*p* = 0.001) with respect to Group C.

## Discussion

This is the first study that examines, using microarray analysis and western blot, the expression of a selection of genes and proteins related to glutamatergic neurotransmission in the TG in rats that were chronically exposed to an enriched environment, or sustained prolonged unilateral whisker trimming, compared to naïve controls. Our major findings can be summarized as follows: (1) in naïve controls, a few genes coding for specific subunits of AMPA and metabotropic receptors (GLUR2 and mGLUR3) and for proteins involved in clustering and stabilization of AMPA receptors (GRIP1 and PICK1) have higher expression in the left than in the right TG. (2) Enrichment led to an overall reduction in expression in the right TG, which was responsible to a large extent for increasing the left–right asymmetry in gene expression observed in controls. This decrease was not reflected in lower protein levels on the right. (3) Vibrissae trimming on the right whiskerpad nearly eliminated the asymmetries in gene expression, owing to a general raise on the right, and to a maintenance or reduction of expression on the left (with the only exception of an increase in Gria4 and Nptx2). Changes in protein levels differed between sides: a marked increase in NMDAR2 in the right TG, and lower levels of GLUR2 and mGLUR3 in the left TG.

### Lateral Asymmetries in Gene Expression and Protein Levels

The existence of lateral asymmetries in the rodent brain has been frequently ignored, or deemed of little relevance compared to the neural bases of obviously lateralized functions in humans. Such lateralizations, which are present in many species and give them adaptive advantages at individual and/or population levels, are likely to arise from an interplay of genetic, epigenetic, and experience-dependent mechanisms (Vallortigara and Rogers, 2005). In the trigeminal system, lateral asymmetries and bilateral effects of unilateral input manipulations have been reported for the barrel cortex (Riddle and Purves, 1995; Machín et al., 2004), and a rightward bias has been found in the whiskers-to-barrel system in rats, which used more efficiently tactile cues with their right-side whiskers and their left somatosensory cortex to learn a novel foraging pattern (LaMendola and Bever, 1997). At peripheral levels of the somatosensory system, bilateral effects in sensory ganglia or the spinal cord or the trigeminal complex have been reported previously, following severe unilateral alterations in sensory input by nerve transection, inflammation or chronic constriction injury (Koltzenburg et al., 1999; Von Banchet et al., 2000; Fukuoka et al., 2001; Samsam et al., 2001; Martin and Avendano, 2009; Dubovy et al., 2013).

At the cellular level, a marked asymmetry was found in the *barrelette* neurons, which are the main neurons in the trigeminal sensory nuclei of the brain stem that relay information from mechanosensory primary afferents to the somatosensory thalamus. These neurons displayed significantly longer dendrites in the left side, and this difference disappeared after chronic whisker trimming on the right side, a manipulation that also brought about a range of bilateral changes in dendrites and spines (Negredo et al., 2009). Moreover, Lagares and Avendaño (2000) had found that the largest, presumably mechanosensory, primary sensory neurons in the TG had on average larger cell bodies on the right side. In addition, it has been reported in humans that sensory nerve conduction velocity differed between the right and left sides (Tan, 1993; Gupta et al., 2008), although no explanation was proposed as to possible neural bases for such asymmetry.

An involvement of glutamate neurotransmission in structural and functional asymmetries has not been examined so far at lower levels of sensory pathways. However, it may occur at higher brain levels, since synapses on spines in the apical dendrite of pyramidal cells in CA1 of either side arising from the left CA3 field were found to be smaller and had higher levels of postsynaptic NMDAR2B, whereas spines contacted by terminals from the right CA3 were larger, richer in GLUR2 AMPA subunit, and poorer in NMDAR2 (Kawakami et al., 2003; Shinohara et al., 2008). Accordingly, this asymmetry had functional consequences, where the left CA3 was able to induce more spike-timing dependent long-term potentiation at CA1 synapses than the right CA3 (Kohl et al., 2011). In the present study we found no significant differences between sides at the protein level for glutamate receptor subunits. However, Gria2, the gene coding for GLUR2, showed higher expression in the left TG, and a parallel asymmetry was found in Pick1 and Grip1, which code for proteins directly implicated in activity-dependent internalization and intracellular stabilization of GLUR2-containing AMPA receptors, events that play important roles in the generation of activity-dependent synaptic plasticity (Takamiya et al., 2008; Terashima et al., 2008).

### Effects of Environmental Enrichment on Glutamate Receptors in the TG

Adult rats display enhanced spontaneous exploratory behavior and active whisking following prolonged exposure to enriched environments (Polley et al., 2004; Valles et al., 2011). These behavioral effects are accompanied by a variety of structural and functional changes in the barrel cortex, such as an increase in cortical thickness and barrelfield volume, expansions of neuronal somata and dendrites, increase in number and size of cortical synapses, and refinements in receptive field size, response selectivity and local field potentials in somatosensory areas (Greenough et al., 1973; Coq and Xerri, 1998; Diamond, 2001; Machín et al., 2004; Devonshire et al., 2010; Landers et al., 2011). A number of transcription factors and IEGs are upregulated in the barrel cortex transiently after short periods of exposure to novel or enriched environments, but these effects were absent or very limited in thalamic and brainstem nuclei relaying trigeminal information (Staiger et al., 2000; Bisler et al., 2002). Extended exposure to enriched environments is known to regulate the differential expression of glutamate receptors in forebrain structures, and, perhaps not coincidentally, some of these effects resemble the changes we found in TG. In particular, there is an increased expression of NMDA receptor subunits NMDAR2B, and NMDAR2A (but not NMDAR1) following enrichment (Tang et al., 2001; Andin et al., 2007; Shum et al., 2007). In our study, the expression of NMDAR2B also tends to be enhanced by enrichment in TG, with a striking lateral asymmetry, also observed in its encoding gene, Grin2B.

The metabotropic receptor subunits in our study belong to groups II and III, which are mainly presynaptic and are involved in reducing neurotransmitter release (Conn and Pin, 1997). Subtypes from either of these groups are present in nearly all DRG and TG neurons, with a predominance of mGLUR8 in neurons of all sizes, followed by mGLUR2/3 in small and medium-sized cells in DRG, and a more general expression in various cell types in TG (Carlton and Hargett, 2007; Boye Larsen et al., 2014). The genes coding for the metabotropic subunits 3, 7, and 8 were downregulated in our study (**Table 2**) on the right side after enrichment, generating a lateral asymmetry that is lacking in controls. This could suggest that glutamate transmission might be enhanced on the right side. Although hard to explain, it is interesting that in young adult rats exposed to enriched environment for 10 weeks, the left barrel cortex (the main target for vibrissal input from the right whiskerpad) showed a 6–9% increase in volume compared to controls kept in standard cages (Machín et al., 2004).

### Effects on Glutamate Receptors in TG of Unilateral Deprivation of Active Touch by Chronic Whisker Trimming

There is a large body of data on the molecular, structural and functional effects that various combinations of active input removal by whisker trimming elicit on the somatosensory cortex (Cheetham et al., 2007; Feldman, 2009; Oberlaender et al., 2012). Data are much scarcer, however, on the long term consequences of these manipulations on the primary sensory neurons innervating the whiskers, or their targets in the trigeminal nuclei of the brain stem. Following unilateral deprivation by chronic whisker trimming, the dendrites of trigeminothalamic *barrelette* neurons in the trigeminal nucleus principalis and of intersubnuclear neurons in the nucleus caudalis undergo bilateral changes in length and distribution of dendritic spines (Negredo et al., 2009; Martin et al., 2014). Bilateral changes were also found in the present study, consisting in an extensive up-regulation of gene expression in the right TG together with a more moderate downregulation on the left side. The genes involved in this change evoked by whisker trimming code for different AMPA, NMDA and metabotropic receptors suggesting that this receptors play active and lasting roles following input removal by whisker trimming.

Amino-3-hydroxy-5-methyl-4-isoxazole propionic acid receptors, in particular those including GLUR1, play a role in enabling experience-dependent and long-term depression in granular and supragranular layers of the barrel cortex in adult mice, since no such depression is observed in GLUR1 knock-out mice (Allen et al., 2003; Wright et al., 2008). Permanent and irreversible deafferentations of the somatic cortex lead to elevated levels of the GLUR1 subunit in monkeys (Mowery et al., 2013, 2015). In contrast, GLUR2 and GLUR3, two subunits that make the AMPA receptor less permeable to Ca^2+^, and which are involved in the insertion and stabilization of AMPA receptors independently of activity, do not differ from controls several weeks after whisker trimming in layer IV of the barrel cortex (Gierdalski et al., 1999) or after permanent deafferentation in somatosensory cortex in monkeys (Mowery et al., 2013, 2015). We found that the genes coding for GLUR1 and GLUR2 (Gria1 and 2) were both upregulated in the deprived right TG. The upregulation of Gria2 on the right ganglion did not translate into increased levels of the protein GLUR2, however, both Gria2 and GLUR2 significantly decreased on the left. Gria4, in contrast, was bilaterally upregulated in TG after unilateral whisker trimming. So far, a similar upregulation of Gria4 has been described in the dorsal horn of the spinal cord 2–4 weeks after sciatic nerve transection (Yang et al., 2004). These findings, together with the likely involvement of GLUR4-containing presynaptic AMPA receptors in modulation of nociception (Willcockson and Valtschanoff, 2008), support the involvement of this subunit in sensory-dependent plasticity processes.

The NMDA receptor subunits NR1 and NR2B display no more than temporary changes in the cortex of adult subjects under conditions of prolonged input deprivation (He et al., 2004; Corson et al., 2009). However, the absence of NMDAR1 leads to an overgrowth of trigeminal primary afferents in the brain stem, which fail to organize into discrete ‘barrelettes’ in the trigeminal nuclei (Lee et al., 2005). In the TG, NMDAR2B increased significantly its levels in the side subjected to chronic deprivation, without a parallel increase in its encoding gene Grin2b. Data are not available for the protein NMDAR1, but its encoding gene (Gria1) also showed higher expression on the deafferented side. NMDAR1 is preferentially expressed in large, mechanosensory primary afferent neurons, and NMDAR2B in small primary afferents, while both subunits have been suggested to be present in satellite cells (Castillo et al., 2013). Therefore, it appears that all cell types directly involved in glutamatergic neurotransmission in the TG might display long-lasting adaptations in their NMDA receptors to input deprivation.

Activation of mGLUR2/3 receptors has been proposed to reduce nociceptive transmission in trigeminal afferents (Boye Larsen et al., 2014). We found that Grm3, the gene encoding mGLUR3, showed a larger basal expression on the left TG, a difference that increased after enrichment, mainly because of a reduced expression on the right TG. Following whisker trimming, in contrast, Grm3 expression rose on the deprived side, without a parallel change in mGLUR3 receptor level, while this protein showed a significant reduction on the contralateral side. It is tempting to speculate that primary sensory neurons enhance the modulatory control that metabotropic receptors exert on excitatory transmission in the deprived side, while matching neurons in the intact side down-regulate these receptors to, putatively, favor input-dependent transmission.

Concerning other metabotropic receptors, in our study the genes encoding mGLUR7 and 8 showed no change (Grm7) or only a moderate decrease on the left side (Grm8). mGLUR7 is expressed in peptidergic small neurons in the DRG, and both the protein and its encoding mRNA are downregulated after sciatic nerve ligation (Li et al., 2012). mGLUR8 is present in both primary sensory neurons and satellite glial cells, and has a high affinity for glutamate, which turns it into a sensitive presynaptic auto- and hetero-receptor that could be activated by local glutamate spillover (Palazzo et al., 2014a). Both subunits regulate glutamate neurotransmission and are involved in reducing nociception at peripheral levels (Govea et al., 2012; Palazzo et al., 2014b) which situate them as appropriate candidates for future sensory-dependent plasticity studies.

### Sensory Experience-Dependent Changes on Glutamate Receptor-Associated Genes and Proteins

Four of the five genes selected under this category (Pick1, Grip1, Dlg4, and Homer1) encode as many PDZ (postsynaptic density 95/discs large/zona occludens) domain-containing proteins that bind to a number of AMPA and NMDA receptor subunits. The fifth corresponds to Nptx2, an IEG that codes for the protein NPTX2, also known as NARP, that is secreted from presynaptic terminals, and induces clustering of GLUR4-containing AMPA receptors.

GRIP1 is a major scaffolding protein that stabilizes in the membrane GluR2/3 containing postsynaptic AMPA receptors. The disruption of GRIP1-GLUR2 interaction is needed for LTD (Kim et al., 2001). Moreover, it has been recently shown that the total amount of GRIP1 and its intracellular distribution is activity- dependent: during neuronal inactivity GRIP1 is reduced, which stabilizes AMPA receptors in the membrane, while under conditions of forced neuronal activity, GRIP1 increases, and removes AMPA from synapses toward the cytoplasm (Tan et al., 2015). PICK1 role in AMPA receptor trafficking is essential for NMDAR-dependent LTD, by its Ca^2+^ dependent capacity to retain AMPA receptors that have been internalized in the first stages of LTD (Terashima et al., 2008; Citri et al., 2010). Both nociceptive and non-nociceptive primary sensory neurons in DRG express presynaptic AMPA receptors containing subunits GLUR1 and GLUR2/3, and most of these receptors concentrate in the central axon terminals of these neurons, strongly inhibiting glutamate release (Lee et al., 2002). While GRIP1 and PICK1 are more abundant postsynaptically, some isoforms of GRIP1 are also present presynaptically at glutamatergic synapses (Charych et al., 2006), and PICK1 is also present in presynaptic membranes, including the active synaptic zone and in the membrane of synaptic vesicles that also contain GluR2 (Haglerod et al., 2009). We found that changes in expression of Grip1 and Pick1 resembled those of Gria2 (that encodes GLUR2) in showing similar lateral asymmetries in controls and a parallel upregulation in the deprived TG.

To our knowledge, Homer1 and Dlg4, as well as their encoded proteins are typically associated to postsynaptic, not presynaptic, membranes, and have not been described up to now in DRG or TG. The expression of these two genes in control TG and the changes in expression associated to enrichment and deprivation is therefore an intriguing finding that needs further investigation.

NPTX2, through its relationship with GLUR4, plays key roles in some learning paradigms and plasticity processes (Tsui et al., 1996; Chang et al., 2010; Johnson et al., 2010; Elbaz et al., 2015). At early stages of development, GLUR4 is critical for the function of synapses, where it is being recruited by weak synaptic activity (Zhu et al., 2000). NARP has been recently reported to be present in small and medium-sized DRG putatively nociceptive neurons, as well as in their terminals in laminae I and II of the dorsal horn of the spinal cord (Miskimon et al., 2014), and the same primary sensory neurons express GLUR4 (Lu et al., 2002). Since in the TG we found that Nptx2 and Gria4 were similarly upregulated bilaterally after unilateral whisker trimming, it could be speculated that a sustained condition of reduced input in the deprived side (perhaps combined with a demand to increase the haptic input contralaterally) were factors involved in the upregulation of those genes. This interpretation, however, should be taken with caution, because NARP is also involved in neural inflammation by regulating macrophage/microglial reaction to various types of pain-inducing peripheral damage (Miskimon et al., 2014), and in DRG GLUR4 is also present in satellite glia (Tachibana et al., 1994).

### Cellular Location of Target Molecules in the TG

A variety of anatomical and molecular techniques have proved conclusively that neuron cell bodies in the DRG or TG are responsible for at least a good part of the gene and protein expression affecting glutamatergic neurotransmission. But three factors complicate this simplified picture considerably. First, many of the glutamatergic-related proteins studied here are enriched in axon terminals, rather than in cell bodies, therefore staying in an undetermined proportion outside the analyzed ganglia. Second, SGC, which intimately surround neuronal cell bodies and regulate many aspects of the neuronal microenvironment, including the trafficking of neurotransmitters (Hanani, 2005), respond with intracellular Ca^2+^ rises to AMPA, NMDA, kainate and mGluR agonists, and express GLUR4, NMDAR1, NR2B and NR3A, mGLUR1, mGluR5 and mGLUR8, while they fail to immunostain for GLUR2/3 (**Supplementary Table S1**; Tachibana et al., 1994; Castillo et al., 2013; Kung et al., 2013; Boye Larsen et al., 2014; Ferrari et al., 2014). However, to our knowledge no data are available as yet concerning the expression of these molecules in DRG or TG in cells other than sensory neurons or SGC. And third, the relationship between transcription and translation is always a complex one, and more so when the target cell has neurites extending far away from the tissue under scrutiny. Presence of glutamate receptors in the soma of DRG or TG neurons has been well established, and presynaptic NMDA, mGLUR and AMPA receptors in primary afferent terminals of the spinal cord are known to be synthesized in the DRG cell bodies, and are mostly transported centrally along the dorsal roots, as has been shown by experimental ligation of dorsal roots or rhizotomies (Araki et al., 1993; Sato et al., 1993; Li et al., 1997; Carlton et al., 2001; Lu et al., 2002, 2003; Boye Larsen et al., 2014). Moreover, recent data suggest the existence of metabotropic receptors in the peripheral terminals of primary sensory neurons contacting peripheral receptors (Watson, 2015). In addition, it is likely that not only proteins but also mRNA exiting the nucleus is dispatched along the axons down to their terminals, where it will translate into protein upon local demand (Alvarez et al., 2000; Crispino et al., 2014). And the presynaptic proteins and even the mRNA in axon terminals might derive, at least in part, from direct transfer from neighboring glial cells (Giuditta et al., 2002). These facts caution against making too hasty inferences of the changes in expression observed in long-term studies.

## Conclusion

For a long time the mature primary sensory neurons were considered relatively passive structures, not playing more than an instrumental and supporting role in the transfer and distribution of sensory information to the central nervous system. Even under conditions of peripheral tissue injury, the role attributed to these neurons dimmed quickly once ‘acute’ pain conditions evolved into a chronic pain disorder (Krames, 2015). In experience-dependent plasticity studies, sensory ganglia have simply been overlooked. Our findings, however, point to the need of investigating in depth the varied responses at the genomic, molecular and cellular levels that sensory ganglia display under changing sensory inputs, which may provide the grounds for adaptation strategies and learning processes.

## Author Contributions

JF-M: Has made substantial contributions to the acquisition, analysis and interpretation of Arrays data for the work, has participated in drafting the article and in revising it critically. IB: has made substantial contributions to the acquisition and analysis of WB data for the work, has participated in revising the article critically. YM: has made substantial contributions to the analysis of data for the work, has participated in revising the article critically. JE: has made substantial contributions to conception and design of the work, has participated in revising the article critically for important intellectual content. PN: has made substantial contributions to conception and design of the work and to the acquisition, analysis and interpretation of data, has participated in drafting the article and in revising it critically for important intellectual content. CA: has made substantial contributions to conception and design of the work and to the analysis and interpretation of data, has participated in drafting the article and in revising it critically for important intellectual content. All authors have given final approval of the version to be submitted and any revised version.

## Conflict of Interest Statement

The authors declare that the research was conducted in the absence of any commercial or financial relationships that could be construed as a potential conflict of interest.

## Footnotes

1 http://www.SABiosciences.com/pcrarraydataanalysis.php

## References

[B1] Akhtar N. D. Land P. W. (1991). Activity-dependent regulation of glutamic acid decarboxylase in the rat barrel cortex: effects of neonatal versus adult sensory deprivation. *J. Comp. Neurol.* 307 200–213. 10.1002/cne.903070204 1713230

[B2] Allen C. B. Celikel T. Feldman D. E. (2003). Long-term depression induced by sensory deprivation during cortical map plasticity in vivo. *Nat. Neurosci.* 6 291–299. 10.1038/nn1012 12577061

[B3] Alvarez J. Giuditta A. Koenig E. (2000). Protein synthesis in axons and terminals: significance for maintenance, plasticity and regulation of phenotype. With a critique of slow transport theory. *Prog. Neurobiol.* 62 1–62. 10.1016/S0301-0082(99)00062-3 10821981

[B4] Andin J. Hallbeck M. Mohammed A. H. Marcusson J. (2007). Influence of environmental enrichment on steady-state mRNA levels for EAA1, AMPA1 and NMDA2A receptor subunits in rat hippocampus. *Brain Res.* 1174 18–27. 10.1016/j.brainres.2007.06.101 17854777

[B5] Araki T. Kenimer J. G. Nishimune A. Sugiyama H. Yoshimura R. Kiyama H. (1993). Identification of the metabotropic glutamate receptor-1 protein in the rat trigeminal ganglion. *Brain Res.* 627 341–344. 10.1016/0006-8993(93)90339-O 8298977

[B6] Bisler S. Schleicher A. Gass P. Stehle J. H. Zilles K. Staiger J. F. (2002). Expression of c-Fos, ICER, Krox-24 and JunB in the whisker-to-barrel pathway of rats: time course of induction upon whisker stimulation by tactile exploration of an enriched environment. *J. Chem. Neuroanat.* 23 187–198. 10.1016/S0891-0618(01)00155-7 11861125

[B7] Bouvier G. Bidoret C. Casado M. Paoletti P. (2015). Presynaptic NMDA receptors: roles and rules. *Neuroscience.* 311 322–340. 10.1016/j.neuroscience.2015.10.033 26597763

[B8] Boye Larsen D. Ingemann Kristensen G. Panchalingam V. Laursen J. C. Norgaard Poulsen J. Skallerup Andersen M. (2014). Investigating the expression of metabotropic glutamate receptors in trigeminal ganglion neurons and satellite glial cells: implications for craniofacial pain. *J. Recept. Signal. Transduct. Res.* 34 261–269. 10.3109/10799893.2014.885049 24495291 PMC4162654

[B9] Brumovsky P. R. (2013). VGLUTs in peripheral neurons and the spinal cord: time for a review. *ISRN Neurol.* 2013:829753 10.1155/2013/829753 PMC3856137 24349795

[B10] Cahusac P. M. Mavulati S. C. (2009). Non-competitive metabotropic glutamate 1 receptor antagonists block activity of slowly adapting type I mechanoreceptor units in the rat sinus hair follicle. *Neuroscience* 163 933–941. 10.1016/j.neuroscience.2009.07.015 19596050

[B11] Carlton S. M. Hargett G. L. (2007). Colocalization of metabotropic glutamate receptors in rat dorsal root ganglion cells. *J. Comp. Neurol.* 501 780–789. 10.1002/cne.21285 17299761

[B12] Carlton S. M. Hargett G. L. Coggeshall R. E. (2001). Localization of metabotropic glutamate receptors 2/3 on primary afferent axons in the rat. *Neuroscience* 105 957–969. 10.1016/S0306-4522(01)00238-X 11530234

[B13] Castillo C. Norcini M. Martin Hernandez L. A. Correa G. Blanck T. J. Recio-Pinto E. (2013). Satellite glia cells in dorsal root ganglia express functional NMDA receptors. *Neuroscience* 240 135–146. 10.1016/j.neuroscience.2013.02.031 23485802

[B14] Chang M. C. Park J. M. Pelkey K. A. Grabenstatter H. L. Xu D. Linden D. J. (2010). Narp regulates homeostatic scaling of excitatory synapses on parvalbumin-expressing interneurons. *Nat. Neurosci.* 13 1090–1097. 10.1038/nn.2621 20729843 PMC2949072

[B15] Charych E. I. Li R. Serwanski D. R. Li X. Miralles C. P. Pinal N. (2006). Identification and characterization of two novel splice forms of GRIP1 in the rat brain. *J. Neurochem.* 97 884–898. 10.1111/j.1471-4159.2006.03795.x 16539648

[B16] Cheetham C. E. Hammond M. S. Edwards C. E. Finnerty G. T. (2007). Sensory experience alters cortical connectivity and synaptic function site specifically. *J. Neurosci.* 27 3456–3465. 10.1523/JNEUROSCI.5143-06.2007 17392462 PMC2043248

[B17] Citri A. Bhattacharyya S. Ma C. Morishita W. Fang S. Rizo J. (2010). Calcium binding to PICK1 is essential for the intracellular retention of AMPA receptors underlying long-term depression. *J. Neurosci.* 30 16437–16452. 10.1523/JNEUROSCI.4478-10.2010 21147983 PMC3004477

[B18] Conn P. J. Pin J. P. (1997). Pharmacology and functions of metabotropic glutamate receptors. *Annu. Rev. Pharmacol. Toxicol.* 37 205–237. 10.1146/annurev.pharmtox.37.1.205 9131252

[B19] Coq J. O. Xerri C. (1998). Environmental enrichment alters organizational features of the forepaw representation in the primary somatosensory cortex of adult rats. *Exp. Brain Res.* 121 191–204. 10.1007/s002210050452 9696389

[B20] Corson J. Nahmani M. Lubarsky K. Badr N. Wright C. Erisir A. (2009). Sensory activity differentially modulates N-methyl-D-aspartate receptor subunits 2A and 2B in cortical layers. *Neuroscience* 163 920–932. 10.1016/j.neuroscience.2009.07.016 19596055 PMC2746878

[B21] Crispino M. Chun J. T. Cefaliello C. Perrone C. C. Giuditta A. (2014). Local gene expression in nerve endings. *Dev. Neurobiol.* 74 279–291. 10.1002/dneu.22109 23853157

[B22] deGroot J. Zhou S. Carlton S. M. (2000). Peripheral glutamate release in the hindpaw following low and high intensity sciatic stimulation. *Neuroreport* 11 497–502. 10.1097/00001756-200002280-00014 10718302

[B23] Devonshire I. M. Dommett E. J. Grandy T. H. Halliday A. C. Greenfield S. A. (2010). Environmental enrichment differentially modifies specific components of sensory-evoked activity in rat barrel cortex as revealed by simultaneous electrophysiological recordings and optical imaging in vivo. *Neuroscience* 170 662–669. 10.1016/j.neuroscience.2010.07.029 20654700

[B24] Diamond M. C. (2001). Response of the brain to enrichment. *An. Acad. Bras. Cienc.* 73 211–220. 10.1590/S0001-37652001000200006 11404783

[B25] Dolan S. Cahusac P. M. (2007). Enhanced short-latency responses in the ventral posterior medial (VPM) thalamic nucleus following whisker trimming in the adult rat. *Physiol. Behav.* 92 500–506. 10.1016/j.physbeh.2007.04.026 17521687

[B26] Dubovy P. Brazda V. Klusakova I. Hradilova-Svizenska I. (2013). Bilateral elevation of interleukin-6 protein and mRNA in both lumbar and cervical dorsal root ganglia following unilateral chronic compression injury of the sciatic nerve. *J. Neuroinflam.* 10:55 10.1186/1742-2094-10-55 PMC3657546 23634725

[B27] Ebner F. F. (2005). *Neural Plasticity in Adult Somatic Sensory-Motor Systems.* Boca Raton, FL: Taylor & Francis/CRC Press.

[B28] Elbaz I. Lerer-Goldshtein T. Okamoto H. Appelbaum L. (2015). Reduced synaptic density and deficient locomotor response in neuronal activity-regulated pentraxin 2a mutant zebrafish. *FASEB J.* 29 1220–1234. 10.1096/fj.14-258350 25466900

[B29] Feldman D. E. (2009). Synaptic mechanisms for plasticity in neocortex. *Annu. Rev. Neurosci.* 32 33–55. 10.1146/annurev.neuro.051508.135516 19400721 PMC3071739

[B30] Ferrari L. F. Lotufo C. M. Araldi D. Rodrigues M. A. Macedo L. P. Ferreira S. H. (2014). Inflammatory sensitization of nociceptors depends on activation of NMDA receptors in DRG satellite cells. *Proc. Natl. Acad. Sci. U.S.A.* 111 18363–18368. 10.1073/pnas.1420601111 25489099 PMC4280647

[B31] Fu S. Y. Gordon T. (1997). The cellular and molecular basis of peripheral nerve regeneration. *Mol. Neurobiol.* 14 67–116. 10.1007/BF02740621 9170101

[B32] Fukuoka T. Kondo E. Dai Y. Hashimoto N. Noguchi K. (2001). Brain-derived neurotrophic factor increases in the uninjured dorsal root ganglion neurons in selective spinal nerve ligation model. *J. Neurosci.* 21 4891–4900. 11425916 10.1523/JNEUROSCI.21-13-04891.2001 PMC6762362

[B33] Gierdalski M. Jablonska B. Smith A. Skangiel-Kramska J. Kossut M. (1999). Deafferentation induced changes in GAD67 and GluR2 mRNA expression in mouse somatosensory cortex. *Mol. Brain Res.* 71 111–119. 10.1016/S0169-328X(99)00153-9 10407193

[B34] Giuditta A. Kaplan B. B. van Minnen J. Alvarez J. Koenig E. (2002). Axonal and presynaptic protein synthesis: new insights into the biology of the neuron. *Trends Neurosci.* 25 400–404. 10.1016/S0166-2236(02)02188-4 12127756

[B35] Govea R. M. Zhou S. Carlton S. M. (2012). Group III metabotropic glutamate receptors and transient receptor potential vanilloid 1 co-localize and interact on nociceptors. *Neuroscience* 217 130–139. 10.1016/j.neuroscience.2012.05.014 22609935 PMC3547613

[B36] Greenough W. T. Volkmar F. R. Juraska J. M. (1973). Effects of rearing complexity on dendritic branching in frontolateral and temporal cortex of the rat. *Exp. Neurol.* 41 371–378. 10.1016/0014-4886(73)90278-1 4126876

[B37] Gupta N. Sanyal S. Babbar R. (2008). Sensory nerve conduction velocity is greater in left handed persons. *Indian J. Physiol. Pharmacol.* 52 189–192. 19130864

[B38] Haglerod C. Kapic A. Boulland J. L. Hussain S. Holen T. Skare O. (2009). Protein interacting with C kinase 1 (PICK1) and GluR2 are associated with presynaptic plasma membrane and vesicles in hippocampal excitatory synapses. *Neuroscience* 158 242–252. 10.1016/j.neuroscience.2008.11.029 19071197

[B39] Hanani M. (2005). Satellite glial cells in sensory ganglia: from form to function. *Brain Res. Brain Res. Rev.* 48 457–476. 10.1016/j.brainresrev.2004.09.001 15914252

[B40] He H. Y. Rasmusson D. D. Quinlan E. M. (2004). Progressive elevations in AMPA and GABAA receptor levels in deafferented somatosensory cortex. *J. Neurochem.* 90 1186–1193. 10.1111/j.1471-4159.2004.02590.x 15312173

[B41] Herrera-Rincon C. Torets C. Sanchez-Jimenez A. Avendano C. Panetsos F. (2012). Chronic electrical stimulation of transected peripheral nerves preserves anatomy and function in the primary somatosensory cortex. *Eur. J. Neurosci.* 36 3679–3690. 10.1111/ejn.12000 23006217

[B42] Johnson A. W. Han S. Blouin A. M. Saini J. Worley P. F. During M. J. (2010). Localized disruption of Narp in medial prefrontal cortex blocks reinforcer devaluation performance. *Learn. Mem.* 17 620–626. 10.1101/lm.1937210 21127001 PMC2998335

[B43] Kaas J. H. Florence S. L. Jain N. (1999). Subcortical contributions to massive cortical reorganizations. *Neuron* 22 657–660. 10.1016/S0896-6273(00)80725-4 10230786

[B44] Kawakami R. Shinohara Y. Kato Y. Sugiyama H. Shigemoto R. Ito I. (2003). Asymmetrical allocation of NMDA receptor epsilon2 subunits in hippocampal circuitry. *Science* 300 990–994. 10.1126/science.1082609 12738868

[B45] Kendziorski C. Irizarry R. A. Chen K. S. Haag J. D. Gould M. N. (2005). On the utility of pooling biological samples in microarray experiments. *Proc. Natl. Acad. Sci. U.S.A.* 102 4252–4257. 10.1073/pnas.0500607102 15755808 PMC552978

[B46] Kim D. S. Lee S. J. Park S. Y. Yoo H. J. Kim S. H. Kim K. J. (2001). Differentially expressed genes in rat dorsal root ganglia following peripheral nerve injury. *Neuroreport* 12 3401–3405. 10.1097/00001756-200110290-00050 11711894

[B47] Kohl M. M. Shipton O. A. Deacon R. M. Rawlins J. N. Deisseroth K. Paulsen O. (2011). Hemisphere-specific optogenetic stimulation reveals left-right asymmetry of hippocampal plasticity. *Nat. Neurosci.* 14 1413–1415. 10.1038/nn.2915 21946328 PMC3754824

[B48] Koltzenburg M. Wall P. D. McMahon S. B. (1999). Does the right side know what the left is doing? *Trends Neurosci.* 22 122–127. 10.1016/S0166-2236(98)01302-2 10199637

[B49] Krames E. S. (2015). The dorsal root ganglion in chronic pain and as a target for neuromodulation: a review. *Neuromodulation* 18 24–32. 10.1111/ner.12247 25354206

[B50] Kung L. H. Gong K. Adedoyin M. Ng J. Bhargava A. Ohara P. T. (2013). Evidence for glutamate as a neuroglial transmitter within sensory ganglia. *PLoS ONE* 8:e68312 10.1371/journal.pone.0068312 PMC3699553 23844184

[B51] Lagares A. Avendaño C. (2000). Lateral asymmetries in the trigeminal ganglion of the male rat. *Brain Res.* 865 202–210. 10.1016/S0006-8993(00)02218-6 10821922

[B52] LaMendola N. P. Bever T. G. (1997). Peripheral and cerebral asymmetries in the rat. *Science* 278 483–486. 10.1126/science.278.5337.483 9334310

[B53] Landers M. S. Knott G. W. Lipp H. P. Poletaeva I. Welker E. (2011). Synapse formation in adult barrel cortex following naturalistic environmental enrichment. *Neuroscience* 199 143–152. 10.1016/j.neuroscience.2011.10.040 22061424

[B54] Lawson S. N. (2005). “The peripheral sensory nervous system: dorsal root ganglion neurons,” in *Peripheral Neuropathy*, eds Dyck P. J. Thomas P. K. (New York, NY: W.B. Saunders), 163–202.

[B55] Lee C. J. Bardoni R. Tong C. K. Engelman H. S. Joseph D. J. Magherini P. C. (2002). Functional expression of AMPA receptors on central terminals of rat dorsal root ganglion neurons and presynaptic inhibition of glutamate release. *Neuron* 35 135–146. 10.1016/S0896-6273(02)00729-8 12123614

[B56] Lee L. J. Lo F. S. Erzurumlu R. S. (2005). NMDA receptor-dependent regulation of axonal and dendritic branching. *J. Neurosci.* 25 2304–2311. 10.1523/JNEUROSCI.4902-04.2005 15745956 PMC3556734

[B57] Li H. Ohishi H. Kinoshita A. Shigemoto R. Nomura S. Mizuno N. (1997). Localization of a metabotropic glutamate receptor, mGluR7, in axon terminals of presumed nociceptive, primary afferent fibers in the superficial layers of the spinal dorsal horn: an electron microscope study in the rat. *Neurosci. Lett.* 223 153–156. 10.1016/S0304-3940(97)13429-2 9080455

[B58] Li J. Y. Wang X. Ji P. T. Li X. F. Guan G. H. Jiang X. S. (2012). Peripheral nerve injury decreases the expression of metabolic glutamate receptor 7 in dorsal root ganglion neurons. *Neurosci. Lett.* 531 52–56. 10.1016/j.neulet.2012.10.014 23085525

[B59] Lu C. R. Hwang S. J. Phend K. D. Rustioni A. Valtschanoff J. G. (2002). Primary afferent terminals in spinal cord express presynaptic AMPA receptors. *J. Neurosci.* 22 9522–9529. 12417676 10.1523/JNEUROSCI.22-21-09522.2002 PMC6758021

[B60] Lu C. R. Hwang S. J. Phend K. D. Rustioni A. Valtschanoff J. G. (2003). Primary afferent terminals that express presynaptic NR1 in rats are mainly from myelinated, mechanosensitive fibers. *J. Comp. Neurol.* 460 191–202. 10.1002/cne.10632 12687684

[B61] Ma Q. P. Hargreaves R. J. (2000). Localization of N-methyl-D-aspartate NR2B subunits on primary sensory neurons that give rise to small-caliber sciatic nerve fibers in rats. *Neuroscience* 101 699–707. 10.1016/S0306-4522(00)00419-X 11113318

[B62] Machín R. Blasco B. Bjugn R. Avendaño C. (2004). The size of the whisker barrel field in adult rats: minimal nondirectional asymmetry and limited modifiability by chronic changes of the sensory input. *Brain Res.* 1025 130–138. 10.1016/j.brainres.2004.07.077 15464753

[B63] Machin R. Perez-Cejuela C. G. Bjugn R. Avendano C. (2006). Effects of long-term sensory deprivation on asymmetric synapses in the whisker barrel field of the adult rat. *Brain Res.* 1107 104–110. 10.1016/j.brainres.2006.05.096 16822483

[B64] Martin Y. B. Avendano C. (2009). Effects of removal of dietary polyunsaturated fatty acids on plasma extravasation and mechanical allodynia in a trigeminal neuropathic pain model. *Mol. Pain* 5 8–17. 10.1186/1744-8069-5-8 19243598 PMC2651866

[B65] Martin Y. B. Negredo P. Villacorta-Atienza J. A. Avendano C. (2014). Trigeminal intersubnuclear neurons: morphometry and input-dependent structural plasticity in adult rats. *J. Comp Neurol.* 522 1597–1617. 10.1002/cne.23494 24178892

[B66] Marvizon J. C. G. McRoberts J. A. Ennes H. S. Song B. B. Wang X. R. Jinton L. (2002). Two N-methyl-D-aspartate receptors in rat dorsal root ganglia with different subunit composition and localization. *J. Comp. Neurol.* 446 325–341. 10.1002/cne.10202 11954032

[B67] Matthews M. R. (1964). Further observations on transneuronal degeneration in the lateral geniculate nucleus of the macaque monkey. *J. Anat.* 98 255–263. 14157007 PMC1261280

[B68] McMahon S. B. Lewin G. Bloom S. R. (1991). The consequences of long-term topical capsaicin application in the rat. *Pain* 44 301–310. 10.1016/0304-3959(91)90101-3 2052400

[B69] Miller K. E. Hoffman E. M. Sutharshan M. Schechter R. (2011). Glutamate pharmacology and metabolism in peripheral primary afferents: physiological and pathophysiological mechanisms. *Pharmacol. Ther.* 130 283–309. 10.1016/j.pharmthera.2011.01.005 21276816 PMC5937940

[B70] Miskimon M. Han S. Lee J. J. Ringkamp M. Wilson M. A. Petralia R. S. (2014). Selective expression of Narp in primary nociceptive neurons: role in microglia/macrophage activation following nerve injury. *J. Neuroimmunol.* 274 86–95. 10.1016/j.jneuroim.2014.06.016 25005116 PMC4152392

[B71] Mowery T. M. Sarin R. M. Kostylev P. V. Garraghty P. E. (2015). Differences in AMPA and GABAA/B receptor subunit expression between the chronically reorganized cortex and brainstem of adult squirrel monkeys. *Brain Res.* 1611 44–55. 10.1016/j.brainres.2015.03.010 25791620 PMC4441862

[B72] Mowery T. M. Walls S. M. Garraghty P. E. (2013). AMPA and GABA(A/B) receptor subunit expression in the cortex of adult squirrel monkeys during peripheral nerve regeneration. *Brain Res.* 1520 80–94. 10.1016/j.brainres.2013.04.032 23643858 PMC4096350

[B73] Negredo P. Martin Y. B. Lagares A. Castro J. Villacorta J. A. Avendano C. (2009). Trigeminothalamic barrelette neurons: natural structural side asymmetries and sensory input-dependent plasticity in adult rats. *Neuroscience* 163 1242–1254. 10.1016/j.neuroscience.2009.07.065 19664693

[B74] Oberlaender M. Ramirez A. Bruno R. M. (2012). Sensory experience restructures thalamocortical axons during adulthood. *Neuron* 74 648–655. 10.1016/j.neuron.2012.03.022 22632723 PMC3564553

[B75] Palazzo E. de Novellis V. Rossi F. Maione S. (2014a). Supraspinal metabotropic glutamate receptor subtype 8: a switch to turn off pain. *Amino Acids* 46 1441–1448. 10.1007/s00726-014-1703-5 24623118

[B76] Palazzo E. Marabese I. de Novellis V. Rossi F. Maione S. (2014b). Supraspinal metabotropic glutamate receptors: a target for pain relief and beyond. *Eur. J. Neurosci.* 39 444–454. 10.1111/ejn.12398 24494684

[B77] Panetsos F. Avendaño C. Negredo P. Castro J. Bonacassa V. (2008). Neural prostheses: electrophysiological and histological evaluation of Central Nervous System alterations due to long-term implants of sieve electrodes to peripheral nerves in cats. *IEEE Trans. Neural. Syst. Rehabil. Eng.* 16 223–232. 10.1109/TNSRE.2008.923707 18586601

[B78] Panetsos F. Nuñez A. Avendaño C. (1995). Local anaesthesia induces immediate receptive field changes in nucleus gracilis and cortex. *Neuroreport* 7 150–152. 10.1097/00001756-199512290-00036 8742439

[B79] Polley D. B. Kvasnak E. Frostig R. D. (2004). Naturalistic experience transforms sensory maps in the adult cortex of caged animals. *Nature* 429 67–71. 10.1038/nature02469 15129281

[B80] Rampon C. Jiang C. H. Dong H. Tang Y. P. Lockhart D. J. Schultz P. G. (2000). Effects of environmental enrichment on gene expression in the brain. *Proc. Natl. Acad. Sci. U.S.A.* 97 12880–12884. 10.1073/pnas.97.23.12880 11070096 PMC18858

[B81] Riddle D. R. Purves D. (1995). Individual variation and lateral asymmetry of the rat primary somatosensory cortex. *J. Neurosci.* 15 4184–4195. 7790904 10.1523/JNEUROSCI.15-06-04184.1995 PMC6577711

[B82] Samsam M. Covenas R. Csillik B. Ahangari R. Yajeya J. Riquelme R. (2001). Depletion of substance P, neurokinin A and calcitonin gene-related peptide from the contralateral and ipsilateral caudal trigeminal nucleus following unilateral electrical stimulation of the trigeminal ganglion; a possible neurophysiological and neuroanatomical link to generalized head pain. *J. Chem. Neuroanat.* 21 161–169. 11312057 10.1016/s0891-0618(01)00088-6

[B83] Sato K. Kiyama H. Park H. T. Tohyama M. (1993). AMPA, KA and NMDA receptors are expressed in the rat DRG neurones. *Neuroreport* 4 1263–1265. 10.1097/00001756-199309000-00013 8219025

[B84] Sawtell N. B. Frenkel M. Y. Philpot B. D. Nakazawa K. Tonegawa S. Bear M. F. (2003). NMDA receptor-dependent ocular dominance plasticity in adult visual cortex. *Neuron* 38 977–985. 10.1016/S0896-6273(03)00323-4 12818182

[B85] Shinohara Y. Hirase H. Watanabe M. Itakura M. Takahashi M. Shigemoto R. (2008). Left-right asymmetry of the hippocampal synapses with differential subunit allocation of glutamate receptors. *Proc. Natl. Acad. Sci. U.S.A.* 105 19498–19503. 10.1073/pnas.0807461105 19052236 PMC2593619

[B86] Shum F. W. Wu L. J. Zhao M. G. Toyoda H. Xu H. Ren M. (2007). Alteration of cingulate long-term plasticity and behavioral sensitization to inflammation by environmental enrichment. *Learn. Mem.* 14 304–312. 10.1101/lm.530607 17522019 PMC2216536

[B87] Staiger J. F. (2006). Immediate-early gene expression in the barrel cortex. *Somatosens Mot. Res.* 23 135–146. 10.1080/08990220601045411 17178549

[B88] Staiger J. F. Bisler S. Schleicher A. Gass P. Stehle J. H. Zilles K. (2000). Exploration of a novel environment leads to the expression of inducible transcription factors in barrel-related columns. *Neuroscience* 99 7–16. 10.1016/S0306-4522(00)00166-4 10924947

[B89] Tachibana M. Wenthold R. J. Morioka H. Petralia R. S. (1994). Light and electron microscopic immunocytochemical localization of AMPA-selective glutamate receptors in the rat spinal cord. *J. Comp. Neurol.* 344 431–454. 10.1002/cne.903440307 8063961

[B90] Takamiya K. Mao L. Huganir R. L. Linden D. J. (2008). The glutamate receptor-interacting protein family of GluR2-binding proteins is required for long-term synaptic depression expression in cerebellar Purkinje cells. *J. Neurosci.* 28 5752–5755. 10.1523/JNEUROSCI.0654-08.2008 18509036 PMC2587083

[B91] Tan H. L. Queenan B. N. Huganir R. L. (2015). GRIP1 is required for homeostatic regulation of AMPAR trafficking. *Proc. Natl. Acad. Sci. U.S.A.* 112 10026–10031. 10.1073/pnas.1512786112 26216979 PMC4538612

[B92] Tan U. (1993). Sensory nerve conduction velocities are higher on the left than the right hand and motor conduction is faster on the right hand than left in right-handed normal subjects. *Int. J. Neurosci.* 73 85–91. 10.3109/00207459308987214 8132422

[B93] Tang Y. P. Wang H. Feng R. Kyin M. Tsien J. Z. (2001). Differential effects of enrichment on learning and memory function in NR2B transgenic mice. *Neuropharmacology* 41 779–790. 10.1016/S0028-3908(01)00122-8 11640933

[B94] Terashima A. Pelkey K. A. Rah J. C. Suh Y. H. Roche K. W. Collingridge G. L. (2008). An essential role for PICK1 in NMDA receptor-dependent bidirectional synaptic plasticity. *Neuron* 57 872–882. 10.1016/j.neuron.2008.01.028 18367088 PMC2336895

[B95] Tropea D. Kreiman G. Lyckman A. Mukherjee S. Yu H. Horng S. (2006). Gene expression changes and molecular pathways mediating activity-dependent plasticity in visual cortex. *Nat. Neurosci.* 9 660–668. 10.1038/nn1689 16633343

[B96] Tsui C. C. Copeland N. G. Gilbert D. J. Jenkins N. A. Barnes C. Worley P. F. (1996). Narp, a novel member of the pentraxin family, promotes neurite outgrowth and is dynamically regulated by neuronal activity. *J. Neurosci.* 16 2463–2478. 8786423 10.1523/JNEUROSCI.16-08-02463.1996 PMC6578758

[B97] Valles A. Boender A. J. Gijsbers S. Haast R. A. Martens G. J. De Weerd P. (2011). Genomewide analysis of rat barrel cortex reveals time- and layer-specific mRNA expression changes related to experience-dependent plasticity. *J. Neurosci.* 31 6140–6158. 10.1523/JNEUROSCI.6514-10.2011 21508239 PMC6632955

[B98] Vallortigara G. Rogers L. J. (2005). Survival with an asymmetrical brain: advantages and disadvantages of cerebral lateralization. *Behav. Brain Sci.* 28 575–589. 10.1017/S0140525X05000105 16209828

[B99] Von Banchet G. S. Petrow P. K. Brauer R. Schaible H. G. (2000). Monoarticular antigen-induced arthritis leads to pronounced bilateral upregulation of the expression of neurokinin 1 and bradykinin 2 receptors in dorsal root ganglion neurons of rats. *Arthritis Res.* 2 424–427. 10.1186/ar121 11056677 PMC17819

[B100] Watson S. (2015). Modulating mechanosensory afferent excitability by an atypical mGluR. *J. Anat.* 227 214–220. 10.1111/joa.12319 26053109 PMC4523323

[B101] Welker E. Soriano E. Van der Loos H. (1989). Plasticity in the barrel cortex of the adult mouse: effects of peripheral deprivation on GAD-immunoreactivity. *Exp. Brain Res.* 74 441–452. 10.1007/BF00247346 2707320

[B102] Willcockson H. Valtschanoff J. (2008). AMPA and NMDA glutamate receptors are found in both peptidergic and non-peptidergic primary afferent neurons in the rat. *Cell Tissue Res.* 334 17–23. 10.1007/s00441-008-0662-0 18679721 PMC2759700

[B103] Wright N. Glazewski S. Hardingham N. Phillips K. Pervolaraki E. Fox K. (2008). Laminar analysis of the role of GluR1 in experience-dependent and synaptic depression in barrel cortex. *Nat. Neurosci.* 11 1140–1142. 10.1038/nn.2188 18776896 PMC3524454

[B104] Xiao H. S. Huang Q. H. Zhang F. X. Bao L. Lu Y. J. Guo C. (2002). Identification of gene expression profile of dorsal root ganglion in the rat peripheral axotomy model of neuropathic pain. *Proc. Natl. Acad. Sci. U.S.A.* 99 8360–8365. 10.1073/pnas.122231899 12060780 PMC123072

[B105] Yang L. A. Zhang F. X. Huang F. Lu Y. J. Li G. D. Bao L. (2004). Peripheral nerve injury induces trans-synaptic modification of channels, receptors and signal pathways in rat dorsal spinal cord. *Eur. J. Neurosci.* 19 871–883. 10.1111/j.0953-816X.2004.03121.x 15009134

[B106] Zhu J. J. Esteban J. A. Hayashi Y. Malinow R. (2000). Postnatal synaptic potentiation: delivery of GluR4-containing AMPA receptors by spontaneous activity. *Nat. Neurosci.* 3 1098–1106. 10.1038/80614 11036266

