# Supplementary material for: Sensory Input-Dependent Changes in Glutamatergic Neurotransmission- Related Genes and Proteins in the Adult Rat Trigeminal Ganglion
Source: Front Mol Neurosci. 2016 Nov 28;9:132. doi: 10.3389/fnmol.2016.00132 (PMC5124698; doi:10.3389/fnmol.2016.00132)
Supplement: Supplementary file 1 [file Table_1.DOCX]

Supplementary Material

SENSORY INPUT-DEPENDENT CHANGES IN GLUTAMATERGIC NEUROTRANSMISSION- RELATED GENES AND PROTEINS IN THE ADULT RAT TRIGEMINAL GANGLION

Julia Fernández-Montoya^1^, Izaskun Buendia^2,4^ , Yasmina B. Martin^1,3^, Javier Egea^2,4^, Pilar Negredo*^1^ and Carlos Avendaño*^1^

*** Correspondence:** Corresponding Author:

Pilar Negredo. Email: pilar.negredo@uam.es

Carlos Avendaño. Email: carlos.avendano@uam.es

# Supplementary Table S1.

**Table S1.** NMDA, AMPA and metabotropic receptor subunit expression in primary sensory neurons and satellite glial cells in DRG/TG.

| **Receptor** | **Neuron** | **SGC** | **References** |
| --- | --- | --- | --- |
| mGluR1 | + | + | Boye Larsen et al., 2014 |
| mGluR2/3 | + | - | Boye Larsen et al., 2014; Sato et al., 1993 |
| mGluR4 | + | ? | Ohishi et al., 1995 |
| mGluR5 | + | + | Abushik et al., 2014 |
| mGluR6 | - | ? | Bardoni, 2013 |
| mGluR7 | + | ? | Li et al., 1997; Ohishi et al., 1995 |
| mGluR8 | + | + | Boye Larsen et al., 2014 |
| GluR1 | + | ? | Lee et al., 2002 |
| GluR2/3 | + | ? | Tachibana et al., 1994; Willcockson and Valtschanoff, 2008 |
| GluR4 | + | + | Kung et al., 2013; Sato et al., 1993;  Tachibana et al., 1994; Willcockson and Valtschanoff, 2008 |
| NR1 | + | + | Ferrari et al., 2014; Marvizon et al., 2002; Sato et al., 1993; Willcockson and Valtschanoff, 2008 |
| NR2a | + | ? | Marvizon et al., 2002 |
| NR2b | + | + | Castillo et al., 2013; Marvizon et al., 2002 |
| NR2c | + | ? | Marvizon et al., 2002 |
| NR2d | + | ? | Marvizon et al., 2002 |
| NR3A | - | + | Ferrari et al., 2014 |
| NR3B | ? | ? | Ferrari et al., 2014 |
